# Association between Obesity and Selected Morbidities: A Study of BRICS Countries

**DOI:** 10.1371/journal.pone.0094433

**Published:** 2014-04-09

**Authors:** Ankita Shukla, Kaushalendra Kumar, Abhishek Singh

**Affiliations:** International Institute for Population Sciences, Deonar, Mumbai, India; University of Washington, United States of America

## Abstract

**Objective:**

Over the past few decades, obesity has reached epidemic proportions, and is a major contributor to the global burden of chronic diseases and disability. There is little evidence on obesity related co-morbidities in BRICS countries. The first objective is to examine the factors associated with overweight and obesity in four of the five BRICS countries (China, India, Russia and South Africa). The second is to examine the linkage of obesity with selected morbidities.

**Methods:**

We used data from the Study on Global Ageing and Adult Health (SAGE) survey conducted by the World Health Organization (WHO) in China, India, Russia and South Africa during 2007–10. The morbidities included in the analysis are Hypertension, Diabetes, Angina, Stroke, Arthritis and Depression.

**Findings:**

The prevalence of obesity was highest in South Africa (35%) followed by Russia (22%), China (5%) and India (3%). The prevalence of obesity was significantly higher in females as compared to males in all the countries. While the wealth quintile was associated with overweight in India and China, engaging in work requiring physical activity was associated with obesity in China and South Africa. Overweight/obesity was positively associated with Hypertension and Diabetes in all the four countries. Obesity was also positively associated with Arthritis and Angina in China, Russia and South Africa. In comparison, overweight/obesity was not associated with Stroke and Depression in any of the four countries.

**Conclusion:**

Obesity was statistically associated with Hypertension, Angina, Diabetes and Arthritis in China, Russia and South Africa. In India, obesity was associated only with Hypertension and Diabetes.

## Introduction

Over the past few decades obesity has emerged as a global health challenge, with every sixth person being obese [1], [2]. In 2008, more than 1.4 billion adults aged 20 or more years were overweight [3]. Of these, 500 million adult men and women were obese. Statistics suggest significant variations in the prevalence of obesity across the globe. The prevalence of obesity ranges from below 5% in China, Japan and African countries to over 75% in urban Samoa [4], [5].

Obesity is known to affect the overall health of a population. According to the World Health Organization (WHO), overweight and obesity are the fifth leading risk for global deaths [3]. Obesity is also found to be associated with a number of non-communicable diseases (NCDs) such as cardiovascular diseases, diabetes, musculoskeletal disorders and some forms of cancers [3]. Statistics indicate that 44% of the diabetes burden, 23% of the heart disease burden and between 7% and 41% of certain cancer burdens can be attributed to overweight and obesity [3].

BRICS is an international association of emerging national economies: Brazil, Russia, India, China, and South Africa. The member countries represent a mix of economies with developing countries like Brazil, India, China and South Africa and a developed country like Russia. The share of China, India, Russia and South Africa together in the total world population is approximately 40%. In terms of population aged 18 years or older, these comprise 41% of the world’s population [6]. These countries are at different stages of demographic and epidemiological transition. Like in many developed countries, obesity is emerging as an important public health problem in BRICS. Economic burden of obesity related NCDs in BRICS is considerable. According to the WHO Report (2005), annual losses in national income from heart disease, stroke and diabetes are estimated at $18 billion in China, $11 billion in Russia, $9 billion in India and $3 billion in Brazil [7]. Furthermore, China is expected to lose $558 billion over the next 10 years in foregone national income due to heart disease, stroke, and diabetes alone. India and Russia are expected to cross $200 billion & $300 billion respectively in the same period [7]. In addition, NCDs and injuries account for an estimated 62% of the total age standardized burden of forgone Disability Adjusted Life Years (DALYs) in India [8].

Several environmental as well as socio-economic and demographic factors are associated with increased risk of overweight and obesity. Age, gender, socio-economic condition, and urban-rural residence are associated with overweight and obesity [9]–[11]. Overweight/obesity are found to be associated with increasing age and female gender [9]–[11]. The other factors that are associated with obesity are urbanization, changing lifestyles, low physical activity, and high calorie intake [12]–[15]. The evidence on association between such factors and obesity in BRICS countries primarily comes from small-scale studies. Moreover, the spread of these risk factors in BRICS countries is relatively less known. In addition, we do not know if all of these factors increase the risk of overweight and obesity in different settings.

Obesity is known to contribute to a variety of health conditions. Studies have shown a significant association between obesity and major health risks such as diabetes, high blood pressure, high cholesterol levels, asthma and arthritis [16], [17]. To our knowledge, there is only one study that has examined the linkage between obesity and hypertension in four of the five BRICS countries [18]. We came across two studies from India [19], [20], four studies from China [21]–[24], one study from South Africa [25], and two studies from Russia [26], [27] that have examined the association between obesity and hypertension/heart disease and have found a significant positive association between them. With reference to studies on obesity and diabetes, we came across three studies only from China [21], [23], [24]. The association between obesity and stroke was examined in a study conducted in China [21], which reported that the obese were more likely to suffer from stroke than normal respondents. Likewise, the association between obesity and arthritis was examined only in a study conducted in rural Russia [26]. It is important to note that, except for Basu & Millett (2013) and Peltzer & Phaswana-Mafuya (2013) [18], [25], all the studies listed above were either based on small-scale datasets (and hence are not representative at the national level) or referred to specific population subgroups. Also, we did not come across any large scale study that examined the association between obesity and depression in these countries.

In view of the issues discussed earlier, the aim of our study is to examine the extent of overweight and obesity in four of the five BRICS countries, namely India, China, South Africa, and Russia. Another aim of the study is to identify the most important factors associated with the increased risk of overweight and obesity in each of these countries. In addition, we examine the association between obesity and selected morbidities – hypertension, diabetes, angina, stroke, arthritis, and depression. Our study is likely to provide more robust and comparable evidence on the association between obesity and selected morbidities in these countries.

## Methods

### Ethics Statement

The study is based on a secondary data set with no identifiable information on the survey participants. This dataset is available in the public domain for research use and hence no formal approval from the institutional review board is required. So, no ethics statement is required for this work. The data can be freely accessed from the WHO website http://apps.who.int/healthinfo/systems/surveydata/index.php/catalog.

### Data

The present study uses data from the Study on Global Ageing and Adult Health (SAGE) surveys implemented by WHO in six countries –China, India, Ghana, Mexico, Russia and South Africa – during 2007–2010. The target population in the SAGE survey is adults, 18 years and older. A multistage stratified clustered sample design was used uniformly in all the countries included in the SAGE. SAGE collected information about self–reported morbidities and health conditions based on interview and health measurement, anthropometric measurements and blood tests [28]. Since our analysis is restricted to BRICS countries, we included China, India, Russia and South Africa in the analysis. Brazil could not be included in our analysis because the SAGE survey was not conducted in Brazil. SAGE interviewed 11,230 adults (18 years or older) in India, 14,811 in China, 4,225 in South Africa, and 4,335 in Russia [28]. Adults for whom weight and height measurements were not available were excluded from the analysis. Hence, the final sample for the analysis was 10,915 for India, 13,898 for China, 3,889 for Russia, and 3,994 for South Africa.

### Variables

In this study, body mass index (BMI) is taken as an indicator of obesity, calculated as weight in kilograms divided by the square of height in meters. Measured weight and height are used to calculate BMI. Respondents are classified as Underweight (BMI <18.5), Normal (18.5≤BMI<25), Overweight (25≤BMI<30) and Obese (BMI≥30) [29].

The inclusion of morbidities in our analysis is based on two criteria – first, the morbidities must be influenced by obesity; and second, information on the morbidities was collected by the SAGE survey. Accordingly, we included hypertension, diabetes, angina, stroke, arthritis, and depression in our analysis. The SAGE survey collected three readings each of systolic and diastolic measures of blood pressure. Any respondent whose systolic measure was 140 or more or whose diastolic measure was 90 or more was coded as ‘suffering from hypertension’. In addition, any respondent who reported as ever been diagnosed with hypertension was also coded as ‘suffering from hypertension’. The rest were coded as ‘not suffering from hypertension’. These cut-offs for hypertension have been recommended and used in a number of studies [19], [28], [30], [31].

For the remaining five morbidities, we used self-reports to code respondents as those having or not having morbidities. The following questions were used to address the remaining five morbidities:

1. Have you ever been diagnosed with diabetes (high blood sugar)? (Yes, No)
2. Have you ever been diagnosed with angina or angina pectoris (a heart disease)? (Yes, No)
3. Have you ever been told by a health professional that you have had a stroke? (Yes, No)
4. Have you ever been diagnosed with/told you have arthritis (or by other names rheumatism or osteoarthritis)? (Yes, No)
5. Have you ever been diagnosed with depression? (Yes, No)

All the morbidities were coded into dummy variables. For example, those reporting ‘yes’ to diabetes were coded as ‘1’ and others were coded as ‘0’.

Since BMI and selected morbidities are known to be affected by other risk factors, several individual and lifestyle factors have been included in the analysis. The variables included in the analysis are: age, gender, schooling, wealth quintiles, place of residence, current smoking status, current alcohol use, and engaged in work that involves physical activity. The wealth quintiles, which are already computed in the SAGE, were generated through a principal components analysis conducted on a set of variables based on the ownership of household assets [32]–[35].

We identified respondents engaged in work that involved physical activity using the following two questions:

1. Does your work involve any type of vigorous-intensity activity that causes large increases in breathing or heart rate [like heavy lifting, digging or chopping wood] for at least 10 minutes continuously? (Yes, No)
2. Does your work involve any type of moderate-intensity activity that causes small increases in breathing or heart rate [such as brisk walking, carrying light loads, cleaning, cooking, or washing clothes] for at least 10 minutes continuously? (Yes, No)

Respondents who reported ‘yes’ to any of the two questions were coded as ‘1’ and the rest were coded as ‘0’.

Two community level variables - percentage of educated respondents in each community and average monthly household expenditure per community - were also included in the analysis.

### Statistical Analysis

Bivariate and multivariate analyses have been used to fulfill the objectives of the paper. Since BMI had four categories (underweight, normal, overweight and obese), we used multinomial logistic regression analysis to examine the factors associated with overweight and obesity. As the selected morbidities were coded as dummy variables, two level random intercept logit models with respondents (level 1) nested within communities (level 2) were fitted to examine the association of obesity with selected morbidities. Appropriate sampling weights were used to control for the complex survey design of the SAGE. Sampling weights are given in the SAGE dataset (See the SAGE reports for details of sampling weight). The analysis presented in the paper was conducted in STATA 12.0.

## Results

The percentage distribution of sample by selected characteristics is shown in Table 1. Among the four countries analyzed, smoking was highest in India (42%) and lowest in Russia (27%). In comparison, drinking alcohol was highest in Russia (41%) and lowest in India (7%). More than three-fourths of the respondents in India and Russia reported that they were engaged in work that involved physical activity. Engagement in work that involved physical activity was reported least in South Africa (52%). Only 24% of the respondents from India and South Africa were 50 years or older. This compares with 36% in China and 40% in Russia. Higher education was more common in the Russian sample; about 86% of the respondents reported that they had high school education or more. This percentage was 24% in India, 27% in China and 28% in South Africa. Rural-urban residence varied considerably across the countries. The percent urban ranged between as low as 31% in the Indian sample to as high as 73% in the Russian sample.

**Table 1:** Percentage distribution of sample by selected characteristics, India, China, Russia and South Africa, 2007–10.

Characteristics	India		China		Russia		South Africa	
	Frequency (N = 10,915)	Percentage	Frequency (N = 13,898)	Percentage	Frequency (N = 3,889)	Percentage	Frequency (N = 3,994)	Percentage
**Currently smoking**								
No	6,685	57.9	10,071	68.4	3,137	73.0	2,946	75.1
Yes	4,230	42.1	3,827	31.6	752	27.0	1,048	24.9
**Currently using alcohol**								
No	10,305	92.6	11,215	77.3	2,869	59.4	3,461	84.7
Yes	610	7.4	2,683	22.8	1,020	40.6	533	15.3
**Physical activity**								
No	2,525	21.5	6,212	36.5	836	15.2	2,209	48.2
Yes	8,388	78.6	7,651	63.5	3,042	84.8	1,692	51.8
**Age group**								
<50 years	4,543	76.0	1,563	64.1	385	59.9	385	75.7
50+	6,372	24.0	12,335	35.9	3,504	40.1	3,836	24.3
**Gender**								
Male	4,249	51.7	6,429	49.6	1,400	44.7	1,704	46.9
Female	6,666	48.3	7,469	50.4	2,489	55.3	2,290	53.1
**Educational level**								
Up to Primary	2,818	60.2	5,159	42.1	368	3.1	1,606	51.0
Secondary	1,357	15.6	3,048	30.7	688	10.9	507	21.0
HS and above	1,813	24.2	2,607	27.2	2,832	86.0	458	28.0
**Wealth quintile**								
Lowest quintile	1,986	20.0	2,755	16.9	738	14.5	688	20.7
2nd quintile	2,087	21.0	2,888	23.8	773	19.4	810	17.7
3rd quintile	2,207	19.1	2,747	17.4	732	15.7	797	19.4
4th quintile	2,293	21.6	2,709	16.3	824	15.9	827	14.5
Top quintile	2,342	18.4	2,799	25.7	822	34.5	872	27.7
**Place of residence**								
Rural	8,164	69.1	7,191	53.6	978	27.1	1,335	30.6
Urban	2,751	31.0	6,707	46.5	2,911	72.9	2,659	69.4

HS refers to higher secondary.

**Figure 1:**
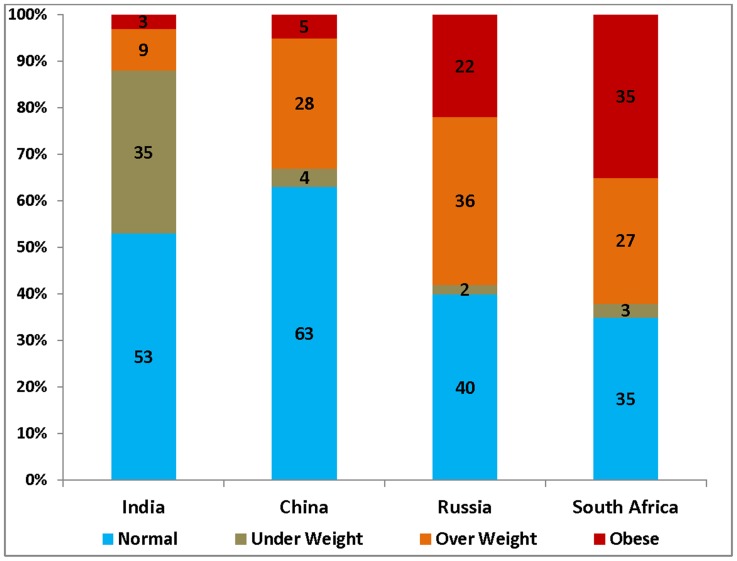
Percentage of population in four BMI categories, India, China, Russia and South Africa, 2007–10.

The prevalence of overweight and obesity in the four countries is shown in Figure 1. The percentage of respondents who were overweight ranged between as low as 9% in India and as high as 36% in Russia. Similarly, the percentage of respondents who were obese ranged between 3% in India and 35% in South Africa. Nearly 5% and 22% of the respondents in China and Russia respectively, were obese.

In multinomial logistic regression, age was associated with overweight and obesity in India, China and Russia (Table 2). 50 years or older respondents were 1.2–2.4 times as likely as respondents below 50 years of age to be overweight than being normal. Likewise, 50 years or older respondents were 1.5–3.7 times as likely as respondents below 50 years of age to be obese than being normal. Gender was statistically associated with overweight and obesity in India and South Africa. In Russia and China, gender was only associated with obesity. Females, when compared to males, were more likely to be overweight or obese than normal in India and South Africa. In Russia and China, females, when compared to males, were more likely to be obese than normal. Wealth was statistically associated with overweight in India and China. However, wealth was not associated with obesity in any of the countries. Similarly, no association was found between educational level and overweight/obesity.

**Table 2:** Relative risk from multinomial logistic regression assessing association between categories of BMI and selected characteristics, India, China, Russia and South Africa, 2007–10.

Characteristics	India			China			Russia			South Africa		
	Underweight	Overweight	Obese	Underweight	Overweight	Obese	Underweight	Overweight	Obese	Underweight	Overweight	Obese
**Currently smoking**												
No®												
Yes	1.38*	0.78*	0.79	1.33*	0.68*	0.75*	1.26	0.58*	0.47*	1.50*	0.68*	0.42*
	(1.25,1.53)	(0.66,0.92)	(0.59,1.06)	(1.06,1.67)	(0.61,0.76)	(0.59,0.95)	(0.56,2.84)	(0.47,0.72)	(0.36,0.61)	(1.03,2.18)	(0.56,0.83)	(0.34,0.51)
**Currently using alcohol**												
No®												
Yes	0.95	1.38	1.25	0.81	0.87*	0.66*	1.20	1.20	1.09	1.61*	0.67*	0.54*
	(0.79,1.14)	(0.99,1.91)	(0.66,2.38)	(0.45,1.02)	(0.78,0.97)	(0.51,0.85)	(0.62,2.73)	(0.99,1.47)	(0.88,1.36)	(1.07,2.43)	(0.52,0.87)	(0.42,0.69)
**Physical activity**												
No®												
Yes	0.82*	1.15	0.85	0.93	0.89*	0.83*	1.90	1.06	1.04	0.51*	0.98	0.76*
	(0.74,0.91)	(0.98,1.35)	(0.66,1.11)	(0.78,1.23)	(0.82,0.97)	(0.70,0.97)	(.72,4.99)	(0.87,1.30)	(0.84,1.29)	(0.35,0.73)	(0.82,1.16)	(0.64,0.90)
**Age**												
Less than 50 years®												
50+	1.09	1.31*	1.47*	0.93	1.21*	1.47*	0.87	2.41*	3.70*	1.18	1.32	1.48*
	(0.99,1.20)	(1.12,1.52)	(1.13,1.91)	(0.70,1.23)	(1.06,1.38)	(1.12,1.94)	(0.37,2.04)	(1.86,3.13)	(2.71,5.07)	(0.82,1.16)	(0.97,1.78)	(1.11,1.98)
**Gender**												
Male®												
Female	1.18*	1.64*	2.72*	1.19	1.02	1.58*	1.50	0.97	2.44*	0.89	1.37*	1.92*
	(1.06,1.31)	(1.38,1.94)	(1.99,3.73)	(0.96,1.48)	(0.92,1.12)	(1.30,1.93)	(0.67,3.35)	(0.80,1.19)	(1.95,3.05)	(0.61,1.29)	(1.13,1.66)	(1.60,2.30)
**Educational** level												
Up to Primary®												
Secondary	0.87	1.16	0.95	0.74*	0.98	1.2	2.88	1.06	1.00	0.37*	1.19	1.14
	(0.76,1.00)	(0.94,1.41)	(0.66,1.38)	(0.58,0.95)	(0.89,1.09)	(0.99,1.45)	(0.62,13.44)	(0.76,1.47)	(0.71,1.41)	(0.16,0.86)	(0.90,1.57)	(0.88,1.49)
HS and above	0.70*	1.19	1.25	0.76	0.91	0.79	1.77	1.29	1.16	0.73	1.26	0.98
	(0.60,0.81)	(0.99,1.43)	(0.91,1.72)	(0.58,1.02)	(0.81,1.02)	(0.62,1.01)	(0.39,3.60)	(0.96,1.73)	(0.86,1.58)	(0.35,1.52)	(0.93,1.72)	(0.73,1.31)
**Wealth quintile**												
Lowest quintile®												
2nd quintile	0.82*	1.49*	0.84	0.65*	1.48*	1.47*	0.76	0.86	1.13	1.07	0.88	1.07
	(0.72,0.93)	(1.06,2.11)	(0.52,1.36)	(0.51,0.82)	(1.30,1.68)	(1.14,1.89)	(0.31,1.86)	(0.66,1.11)	(0.86,1.49)	(0.67,1.72)	(0.67,1.17)	(0.83,1.40)
3rd quintile	0.62*	2.34*	0.94	0.67*	1.55*	1.14	0.43	0.98	1.11	0.94	1.27	1.3
	(0.54,0.70)	(1.70,3.21)	(0.60,1.48)	(0.51,0.88)	(1.33,1.78)	(0.85,1.53)	(1.44,1.30)	(0.75,1.28)	(0.83,1.48)	(0.55,1.60)	(0.95,1.69)	(0.99,1.72)
4th quintile	0.48*	3.53*	1.17	0.48*	1.50*	0.8	0.68	1.13	1.70*	0.7	1.58*	1.90*
	(0.42,0.56)	(2.60,4.79)	(0.76,1.81)	(0.34,0.67)	(1.28,1.77)	(0.58,1.12)	(0.26,1.80)	(0.86,1.48)	(1.27,2.28)	(0.37,1.33)	(1.15,2.16)	(1.41,2.56)
Top quintile	0.36*	5.27*	2.65*	0.47*	1.72*	0.99	0.49	0.99	1.51*	0.65	1.88*	2.42*
	(0.30,0.42)	(3.83,7.26)	(1.71,4.10)	(0.33,0.68)	(1.46,2.04)	(0.71,1.38)	(0.17,1.43)	(0.75,1.30)	(1.12,2.03)	(0.31,1.36)	(1.34,2.64)	(1.75,3.32)
**Place of residence**												
Rural®												
Urban	0.80*	1.24*	1.06	1.1	1.12	1.81*	1.17	0.93	0.50*	1.21	0.99	1.17
	(0.71,0.91)	(1.07,1.44)	(0.80,1.37)	(0.84,1.42)	(0.99,1.25)	(1.44,2.28)	(0.52,2.64)	(0.76,1.15)	(0.40,0.62)	(0.81,1.81)	(0.81,1.23)	(0.96,1.43)

*p<0.05, ®indicates reference category, HS refers to higher secondary, Marital status is controlled in the model.

Smoking was also associated with both overweight and obesity in China, South Africa and Russia (Table 2). Smokers were less likely than non-smokers to be either overweight (odds ratio: 0.58–0.68) or obese (odds ratio: 0.42–0.75) than normal. Drinking alcohol was associated with overweight and obesity in China and South Africa. While engaging in work that involved physical activity was associated with both overweight and obesity in China, it was associated only with obesity in South Africa. Respondents whose work involved physical activity were less likely to be obese than respondents whose work did not involve physical activity in these countries (Odds ratio for China –0.83; Odds ratio for South Africa –0.76).

Table 3 shows the prevalence of selected morbidities in India, China, South Africa and Russia. The prevalence of hypertension was highest in South Africa (51%) and lowest in India (26%). The prevalence of diabetes ranged between 3% in India and China to 4% in Russia. Nearly 16% of the respondents in Russia reported angina compared to only 3%-4% in India, China and South Africa. Of the four countries considered in the analysis, the prevalence of arthritis was the highest in Russia (19%) and the least in South Africa (8%). The prevalence of depression ranged between 0.4% in China and 5% in South Africa.

**Table 3:** Prevalence of selected morbidities by BMI categories, India, China, Russia and South Africa, 2007–10.

BMI	Arthritis	Hypertension	Diabetes	Angina	Stroke	Depression
***India***						
Normal	9.2	26.6	2.9	3.3	1.2	3.4
Underweight	7.5	20.2	2.1	2.9	0.6	2.7
Overweight	17.6	42.8	8.1	3.6	1.1	6.3
Obese	8.9	43.4	4.9	3.1	0.6	2.9
Total	9.4	26.3	3.2	3.1	1.0	3.4
***China***						
Normal	13.3	36.6	1.9	3.4	0.9	0.2
Underweight	10.0	27.9	0.7	3.3	1.7	0.2
Overweight	15.5	55.9	5.0	5.3	1.3	0.6
Obese	22.9	70.0	10.8	7.5	1.1	1.6
Total	14.3	43.2	3.2	4.1	1.1	0.4
***Russia***						
Normal	14.5	22.9	2.3	10.2	2.2	2.7
Underweight	1.2	10.5	10.1	4.1	0.2	9.0
Overweight	14.0	45.7	3.4	15.3	2.3	2.4
Obese	34.8	73.1	6.8	30.6	2.7	3.1
Total	18.6	42.0	3.8	16.4	2.3	2.8
***South Africa***						
Normal	3.8	35.0	1.4	1.1	0.9	6.3
Underweight	5.2	47.0	0.6	0.2	3.1	0.5
Overweight	7.6	48.1	3.3	2.2	1.5	1.0
Obese	13.7	70.6	5.3	4.9	1.7	6.8
**Total**	8.4	51.4	3.3	2.7	1.4	4.9

Results, adjusted for selected demographic, socioeconomic, lifestyle and residence related characteristics, suggest that obesity was associated with arthritis, hypertension, diabetes, and angina in China, Russia and South Africa (Table 4). Obese respondents were 1.4–2.5 times as likely as normal respondents to report arthritis in these countries. Likewise, obese respondents were 1.9–3.5 times as likely as normal respondents to have hypertension. Similarly, obese respondents were 2.1–3.0 times more likely to report diabetes than the normal respondents. Obese were also more likely to report angina than normal respondents. In India, obesity was only associated with hypertension and diabetes – obese respondents were significantly more likely than the normal respondents to have hypertension or diabetes. Obesity was not associated with either stroke or depression in any of the selected countries.

**Table 4:** Odds ratios and standard errors from two-level random intercept logistic regression models assessing association between BMI levels and selected morbidities, India, China, Russia and South Africa, 2007–10.

BMI	Arthritis	Hypertension	Diabetes	Angina	Stroke	Depression
***India***						
Normal®						
Underweight	0.76* (0.06)	0.67* (.030)	0.51* (0.07)	0.92 (0.11)	0.82 (0.16)	0.88 (0.11)
Overweight	1.48* (0.14)	1.71* (0.12)	1.53* (0.19)	1.15 (0.18)	0.82 (0.21)	0.99 (0.20)
Obese	1.23 (0.21)	1.96* (0.23)	2.36* (0.45)	1.11 (0.31)	0.71 (0.38)	1.60 (0.55)
PSU level variance	0.81 (0.10)	0.11* (0.02)	0.27* (0.09)	0.57* (0.15)	0.38* (0.16)	2.36* (0.38)
VPC	0.20	0.03	0.08	0.15	0.10	0.42
***China***						
Normal®						
Underweight	0.90 (0.10)	0.54* (0.05)	0.35* (0.11)	1.04 (0.19)	0.67 (0.20)	0.59 (0.63)
Overweight	1.16* (0.06)	2.12* (0.09)	1.57* (0.12)	1.13 (0.08)	1.20 (.014)	1.06 (0.40)
Obese	1.45* (0.13)	3.03* (0.28)	2.29* (0.30)	1.44* (0.18)	1.09 (0.24)	1.22 (0.78)
PSU level variance	0.12* (0.03)	0.10* (0.02)	0.10* (0.05)	0.48* (0.12)	0.30 (0.10)	0.18* (0.22)
VPC	0.03	0.03	0.03	0.13	0.08	0.05
***Russia***						
Normal®						
Underweight	0.28* (0.16)	0.47* (0.18)	1.40 (1.05)	0.56 (0.27)	1.40 (1.11)	0.45 (0.49)
Overweight	1.10 (0.11)	1.73* (0.16)	1.87* (0.38)	1.14 (0.12)	1.12 (0.22)	0.66* (0.14)
Obese	1.81* (0.12)	3.47* (0.39)	2.96* (0.58)	1.66* (0.18)	1.37 (0.28)	0.73 (0.16)
PSU level variance	0.53* (0.12)	0.21* (0.07)	0.16* (0.11)	0.67 *(0.15)	0.50 (0.22)	0.68 (0.27)
VPC	0.14	0.03	0.05	0.17	0.13	0.17
***South Africa***						
Normal®						
Underweight	1.68* (0.42)	0.78 (0.15)	0.45 (0.29)	0.45 (0.29)	1.48 (0.65)	0.76 (0.49)
Overweight	1.47* (0.20)	1.53* (0.16)	1.81* (0.37)	1.09 (0.23)	0.97 (0.27)	0.93 (0.28)
Obese	2.47* (0.31)	1.89* (0.20)	2.12* (0.40)	1.67* (0.31)	1.20 (0.30)	1.14 (0.31)
PSU level variance	0.47 (0.09)	0.35* (0.08)	0.47* (0.14)	0.48* (0.17)	0.12 (0.20)	0.01* (0.01)
VPC	0.13	0.10	0.13	0.13	0.03	0.00

*p<0.05.

®indicates reference category.

Marital status, age, gender, alcohol consumption, physical activity, smoking, educational level, wealth quintiles and place of residence are controlled in the model.

VPC (Variance partition coefficient) is the proportion of total residual variance which is attributable to the community characteristics.

Like obesity, overweight was also associated with hypertension and diabetes in all the four selected countries. Overweight respondents were 1.5–2.1 times more likely than normal respondents to report hypertension. Likewise, overweight respondents were 1.5–1.9 times more likely than normal respondents to report diabetes. The association between overweight and arthritis was significant in India, China and South Africa (odds ratio: 1.5, 1.2 and 1.5 in India, China and South Africa respectively). Around 0.2% - 42% of residual variation in the chances of suffering from selected morbidities in selected countries was attributable to differences in the community characteristics.

## Discussion

Overweight and obesity varied considerably across four of the five BRICS countries included in the analysis. The percentage overweight ranged between as low as 9% in India to as high as 37% in Russia. The percentage of obese ranged between as low as 3% in India to as high as 35% in South Africa. The prevalence of selected morbidities also varied considerably across the four countries. The prevalence of hypertension, angina, stroke and diabetes were considerably higher in Russia compared to the other three countries included in the analysis. To account for the differences in the age-structure of the sampled respondents in four countries, we also estimated age-standardized prevalence for selected morbidities in each country. The age-standardized rates also suggested a considerably high burden of hypertension, angina, stroke and diabetes in Russia compared to the other three countries (results not shown).

Overweight/obesity was found to be associated with hypertension and diabetes in all the four countries. Our findings confirm the findings of the three small-scale studies conducted on a similar topic in China [21], [23], [24]. Obese respondents were also more likely to report arthritis in China, South Africa and Russia. In contrast to our findings, Kalichman et al. (2006) found no association between obesity and arthritis in rural Russia [26]. Angina was also more likely to be reported by obese respondents in China, South Africa and Russia. Unlike earlier studies, stroke was not associated with overweight/obesity in our study. The relationship between overweight/obesity and stroke might get affected by the way in which information was collected about stroke in the SAGE. The question on stroke in the SAGE did not distinguish between ischemic stroke and hemorrhagic stroke. A recent meta-analysis of prospective studies found increased risk of ischemic stroke in both overweight and obese individuals [36]. Hemorrhagic stroke however was not related to overweight/obesity. Depression was also not associated with overweight/obesity in our study.

One of the strengths of the SAGE survey is that it also allows for estimation of symptom based prevalence of selected morbidities such as angina, arthritis and depression. To examine whether the association between overweight/obesity and these morbidities is robust, we also estimated binary logistic regressions using symptom based prevalence as the dependent variable. The associations of overweight/obesity with arthritis and depression were similar to those obtained in self-reported prevalence. When we used the symptom based prevalence of angina, the association between overweight/obesity and angina became statistically significant even in India (results not shown). In India, the relationship between overweight/obesity and angina, in self-reported diagnosis, might get affected by the biases related to health system access. Self-reported diagnoses could reflect systematic over or under reporting of NCDs due to biases related to health system access [37].

The strengths and limitations of our study must be noted. One of the strengths of our study is the use of a large-scale population based dataset for examining the consequences of overweight/obesity in four of the five BRICS countries. Our study, perhaps for the first time, has provided robust and comparable evidence on the association between overweight/obesity and selected morbidities in BRICS countries. The large sample sizes allowed us to control for a number of pertinent covariates in the multivariate statistical models. The limitations of our study are that our study only establishes an association between overweight/obesity and selected morbidities and does not provide any evidence for causality. Second, the choice of control variables was limited to only those variables that were available for each of the four countries considered in the analysis.

To conclude, overweight/obesity was found to be an important risk factor for hypertension, angina, diabetes and arthritis. It is thus important that public health programs focus their attention towards the growing epidemic of obesity before it reaches alarming levels. Interestingly, obesity is preventable [2]. Obesity prevention policies should focus on facilitating key behaviors like choosing healthier food, limiting unhealthy food, increasing physical activity, improving sleep, reducing stress, etc. (http://www.hsph.harvard.edu/obesity-prevention-source/obesity-prevention). To achieve these, action is needed at multiple levels – from governments and industries, communities and neighborhoods, schools and workplaces, and individual and families [38]–[40]. For example, to curb consumption of sugary drinks, governments could levy heavy taxes. A recent Indian study has shown that sustained high tax rates on sugar-sweetened beverages could mitigate rising obesity and type-2 diabetes [18]. Schools can increase the recess time and promote dietary education and physical activity. Although evidence on how to prevent obesity is growing, there is a dearth of obesity prevention studies in low- and middle-income countries [41]. Hence future studies must examine the impact of obesity prevention strategies in low- and middle-income countries.
